# Theoretical studies on the two-photon absorption of II–VI semiconductor nano clusters

**DOI:** 10.1038/s41598-021-04203-w

**Published:** 2022-01-07

**Authors:** Deyang Yu, YangYang Hu, Guiling Zhang, Weiqi Li, Yongyuan Jiang

**Affiliations:** 1 School of Materials Science and Chemical Engineering, Harbin University of Science and Technology, Harbin, 150080 China; 2 Key Laboratory of Green Chemical Technology of College of Heilongjiang Province, Harbin University of Science and Technology, Harbin, 150080 China; 3 School of Physics, Harbin Institute of Technology, Harbin, 150001 China; 4 Key Lab of Micro-Optics and Photonic Technology of Heilongjiang Province, Harbin, 150001 China; 5 State Key Laboratory of Intense Pulsed Radiation Simulation and Effect, Xi’an 710024, China

**Keywords:** Optical physics, Atomic and molecular physics

## Abstract

Semiconductor clusters, Zn_n_O_n_, Zn_n_S_n_, and Cd_n_S_n_ (n = 2–8), were optimized and the corresponding stable structures were acquired. The symmetry, bond length, bond angle, and energy gap between HOMO and LUMO were analyzed. According to reasonable calculation and comparative analysis for small clusters Zn_2_O_2_, Zn_2_S_2_, and Cd_2_S_2_, an effective method based on density function theory (DFT) and basis set which lay the foundation for the calculation of the large clusters have been obtained. The two-photon absorption (TPA) results show that for the nano clusters with planar configuration, sizes play important role on the TPA cross section, while symmetries determine the TPA cross section under circumstance of 3D stable structures. All our conclusions provide theoretical support for the development of related experiments.

## Introduction

Due to the three-dimensional (3D) quantum confinement effect, II–VI semiconductor clusters are proved to have significant nonlinear optical (NLO) properties and are widely used in physics, chemistry, and biomedical engineering^1,2^. Compared with bulk materials, nano clusters have greater two-photon absorption (TPA) cross section and nonlinear refractivity, and the absorption peak and absorption coefficient are closely related with their sizes, structures, specific surface area, surface defect, ligand type, way of bonding and so forth^3–8^. So far, it is possible to synthesize II–VI group semiconductor quantum dots using collochemistry method, and the researches of NLO properties mostly focuses on the second/third order susceptibility. Amit D. Lad et al. used the open-hole Z-scanning technique to investigate the third order NLO properties of ZnSe with different sizes^9^. It shows that the absorption cross section increased with decreased diameter of quantum dots. Xiaobo Feng and Wei Ji investigated the TPA of nano semiconductor crystal with different shapes and sizes^10^. They compared the absorption cross section of CdS nano sphere (4.45 nm in diameter) and CdS nano cylinder (4.4 nm in diameter and 43 nm in length). It turns out that CdS nano sphere had an absorption cross section of 10^3^ GM and 10^4^ ~ 10^5^ GM for CdS nano cylinder, which can be explained that the reduced symmetry of nano cylinder resulted in the division of energy state, and this leads to an increased density of energy state.

Although the great improvements in the synthesis of the semiconductor nano clusters^11–14^, it is still difficult to obtain samples with uniform sizes and controllable shapes experimentally, further realize the measurement of optical properties of nano clusters with the same size and morphology. Fortunately, theoretical calculations can fill the gap in this regard, such as the prediction for experiments, and the analysis and study of the experimental results. Up to now, the investigation of mechanism and computation method of semiconductor nano clusters’ spectra has made some achievable breakthrough^15–18^. There are studies about relatively stable semiconductor quantum dots and their electronic properties using first-principles theory, and it has been explained how the stability of system is determined by the width of energy gap between conduction band and valence band, as well as how the energy gap is determined by the system’s bonding energy. Perry et al. utilized experiments combined with theoretical calculations to systematically compare the one-photon and two-photon spectroscopy of CdSe nano clusters and organic molecules^19^. The results pointed out that semiconductor clusters over 3 nm had NLO properties with bulk materials and could be described using effective mass model.

Despite the reported TPA from experiments and theories, there is still rare researches on the structural information and evolution rules of semiconductor nano clusters, and the existing studies still have much stochasticity. The lack of understanding the relevancy of the cluster structures on its properties makes it necessary to establish a comprehensive theoretical analysis. Aimed at exploring the correlation between the TPA of small nano clusters (Zn_n_O_n_, Zn_n_S_n_, and Cd_n_S_n_, n = 2–8) and their structures, the present paper are organized as follows: (1) the choice of the appropriate density functional method and basis set for predicting the NLO response of the semiconductor nano clusters; (2) the rules of changing TPA cross section with structures. This will be of great importance to the research of the semiconductor nano clusters, because on one hand, an effective method and basis set for the calculation of the structures of large clusters will be obtained. And on the other hand, the effects of cluster sizes and structure on the TPA will be studied, the rules will be summarized and explained. They all provide theoretical support for the development of related experiments.

## Computational method

In order to find out the most suitable methods based on density functional theory (DFT) for calculating the TPA cross section of the semiconductor nano clusters, eight different methods were chosen for the exploration, including local density approximation functional SVWN^20–23^; generalized gradient approximation BPW91^24–29^; hybrid functional B3LYP, DBLYP, X3LYP, and BHandLYP^30–33^; long range correct functional CAM-B3LYP^34^; double hybrid functional B2PLYP^35^. By using the above methods, the first four transition energies of Zn_2_O_2_ and Zn_2_S_2_ were calculated and compared with that obtained from the high-precision coupled cluster method including singles and doubles fully (CCSD)^36–39^, and a proper DFT method was chosen. The all-electronic basis set 6-31G* was used for the calculation of Zn with small atomic numbers. Due to the large atomic number of Cd, the all-electron basis set is not applicable, so the pseudopotential basis set is considered. The transition energies for the first six excited states of Cd_2_S_2_ were calculated using three different pseudopotential basis sets cc-pVDZ-pp, LANL2DZ, and SDD as well as the aug-cc-pVDZ-pp basis set, and the suitable pseudopotential basis sets to be used in the calculation of larger systems was selected.

Different structures of II–VI group semiconductor nano clusters of Zn_n_O_n_, Zn_n_S_n_, and Cd_n_S_n_, n = 2–8 were generated, and the nano clusters which contained 4 to 16 atoms were optimized by Gaussian09 software^40^. The TPA cross-section (*δ*_TPA_) were evaluated by means of calculating the two-photon transition moment matrix elements (*S*_αβ_) in the Dalton package^41–45^. For two-photon absorption, the *S*_αβ_ expressed as

1 $${\mathrm{S}}_{\alpha \beta }=\sum_{n}\left[\frac{\langle 0|{\mu }_{\alpha }|n\rangle \langle n|{\mu }_{\beta }|f\rangle }{{\omega }_{n}-\frac{{\omega }_{f}}{2}}+\frac{\langle 0|{\mu }_{\beta }|n\rangle \langle n|{\mu }_{\alpha }|f\rangle }{{\omega }_{n}-\frac{{\omega }_{f}}{2}}\right]$$

For the absorption of two photons of identical energy, where *n* ranges from the ground state 0 to the final excited state *f*. The calculated *S*_αβ_ can then be used to obtain the *δ*_TPA_, as shown in Eq. (2)

2 $${\delta }_{TPA}=\frac{1}{30}\sum_{\alpha \beta }F{S}_{\alpha \alpha }{S}_{\beta \beta }+G{S}_{\alpha \beta }{S}_{\alpha \beta }+H{S}_{\alpha \beta }{S}_{\beta \alpha }$$

where the summations are performed over the molecular axes (i.e., *x*, *y*, and *z* in Cartesian coordinates), and *F*, *G*, and *H* depend on the polarization vectors of the incoming photons. Assuming that the incident radiation is linearly polarized monochromatic light, the transition moment for TPA (in atomic units) is

3 $${\delta }_{TPA}=\frac{1}{30}\sum_{\alpha , \beta }2{S}_{\alpha \alpha }{S}_{ \beta \beta }^{*}+4{S}_{\alpha \beta }{S}_{ \beta \beta }^{*}$$

In view of the relation to the experimental measurements, the *δ*_TPA_ is usually expressed in terms of Göppert-Mayer (GM) units, where 1 GM is 10^–50^ cm^4^ s photon^−1^ molecule^−1^. As a result, the relationship between the macroscopic TPA cross section in GM (*σ*_TPA_) and the immediate computation output in atomic units (*δ*_TPA_) is given by

4 $${\upsigma }_{TPA}=\frac{4{\pi }^{2}{a}_{0}^{5}\alpha }{15c}\frac{{\omega }_{f}^{2}}{\Gamma}{\delta }_{TPA}$$

where *α* is the fine structure constant, *a*_0_ is the Bohr radius, *c* is the speed of light, *ω*_*f*_ is the excitation energy for the 0 → *f* transition, and *Γ* is the broadening width.

## Results and discussion

**Figure 1:**
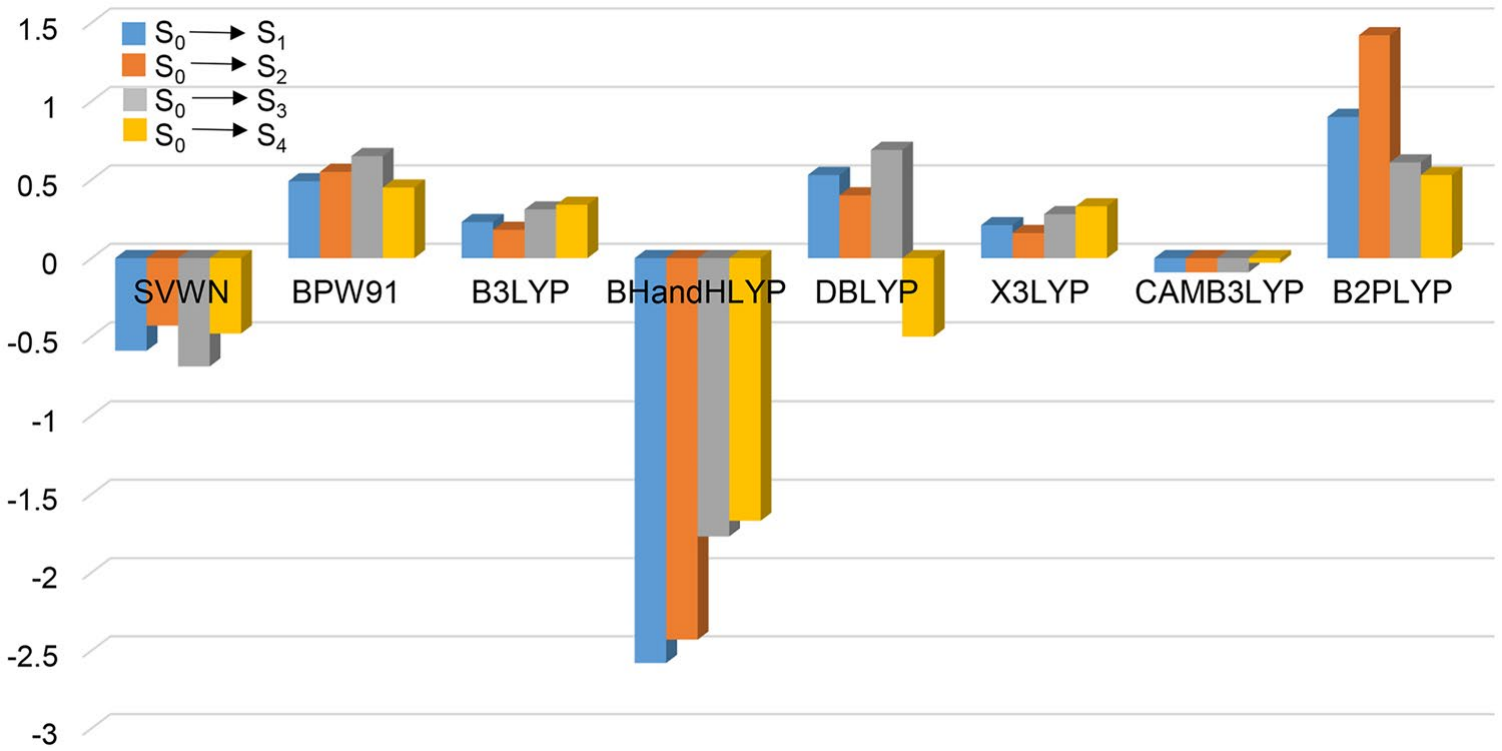
Errors for the transition energies of the first four excited states between CCSD and the eight different DFT methods for Zn_2_O_2._

Errors of the first four excited state transition energies between CCSD and the eight DFT methods for Zn_2_O_2_ are shown in Table 1 and Fig. 1. It can be clearly seen from Table 1 that the errors for the transition energies of the first four excited states of Zn_2_O_2_ between CAM-B3LYP and CCSD are − 0.09, − 0.09, − 0.09, and − 0.03, much smaller than the other seven DFT methods. The largest error comes from BHandLYP with − 2.58, − 2.43, − 1.77, and − 1.67, respectively. According to the above results analysis, the accuracy of the eight DFT methods sort from the largest to the smallest are: CAM-B3LYP > X3LYP > B3LYP > DBLYP > BPW91 > SVWN > B2LYP > BHandLYP.

**Table 1:** Errors for the transition energies of the first four excited states between CCSD and the eight different DFT methods for Zn_2_O_2._

Methods\excited state	S_0_ → S_1_	S_0_ → S_2_	S_0_ → S_3_	S_0_ → S_4_
SVWN	− 0.59	− 0.43	− 0.69	− 0.48
BPW91	0.49	0.55	0.65	0.45
B3LYP	0.23	0.18	0.31	0.34
BHandHLYP	− 2.58	− 2.43	− 1.77	− 1.67
DBLYP	0.53	0.40	0.69	− 0.50
X3LYP	0.21	0.16	0.28	0.33
CAMB3LYP	− 0.09	− 0.09	− 0.09	− 0.03
B2PLYP	0.90	1.42	0.61	0.53

**Figure 2:**
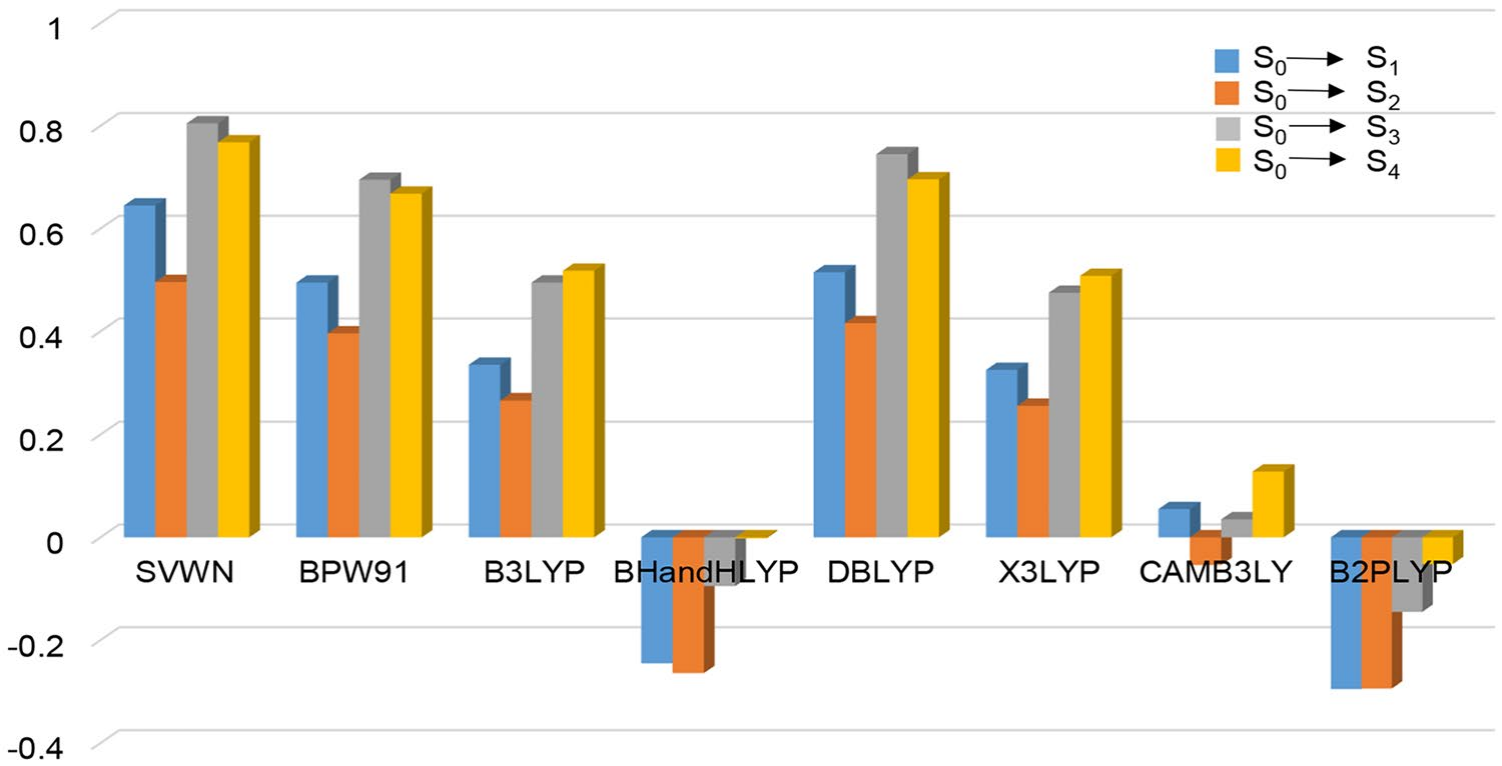
Errors for the transition energies of the first four excited states between CCSD and the eight different DFT methods for Zn_2_S_2._

Errors of the first four excited state transition energies between CCSD and the eight DFT methods for Zn_2_S_2_ are shown in Table 2 and Fig. 2. It can be seen from the data that errors for the transition energies of the first four excited states of Zn_2_S_2_ between CAM-B3LYP and CCSD are also the smallest (0.06, − 0.05, 0.04, and 0.13, respectively). The maximal errors appear in SVWN with the values 0.65, 0.50, 0.81, and 0.77, respectively. The accuracy of the eight DFT methods sort from largest to smallest are: CAM-B3LYP > BHandLYP > B2LYP > X3LYP > B3LYP > BPW91 > DBLYP > SVWN. In summary, CAM-B3LYP is more suitable for the calculating of the TPA for the II–VI semiconductor clusters, and it will be selected to predict the *σ*_TPA_ value of Zn_n_O_n_, Zn_n_S_n_, and Cd_n_S_n_, n = 2–8 in present work.

**Table 2:** Errors for the transition energies of the first four excited states between CCSD and the eight different DFT methods for Zn_2_S_2._

Methods\excited state	S_0_ → S_1_	S_0_ → S_2_	S_0_ → S_3_	S_0_ → S_4_
SVWN	0.65	0.50	0.81	0.77
BPW91	0.50	0.40	0.70	0.67
B3LYP	0.34	0.27	0.50	0.52
BHandHLYP	− 0.25	− 0.26	− 0.1	− 0.00
DBLYP	0.52	0.42	0.75	0.70
X3LYP	0.33	0.26	0.48	0.51
CAMB3LYP	0.06	− 0.05	0.04	0.13
B2PLYP	− 0.30	− 0.29	− 0.15	− 0.05

**Figure 3:**
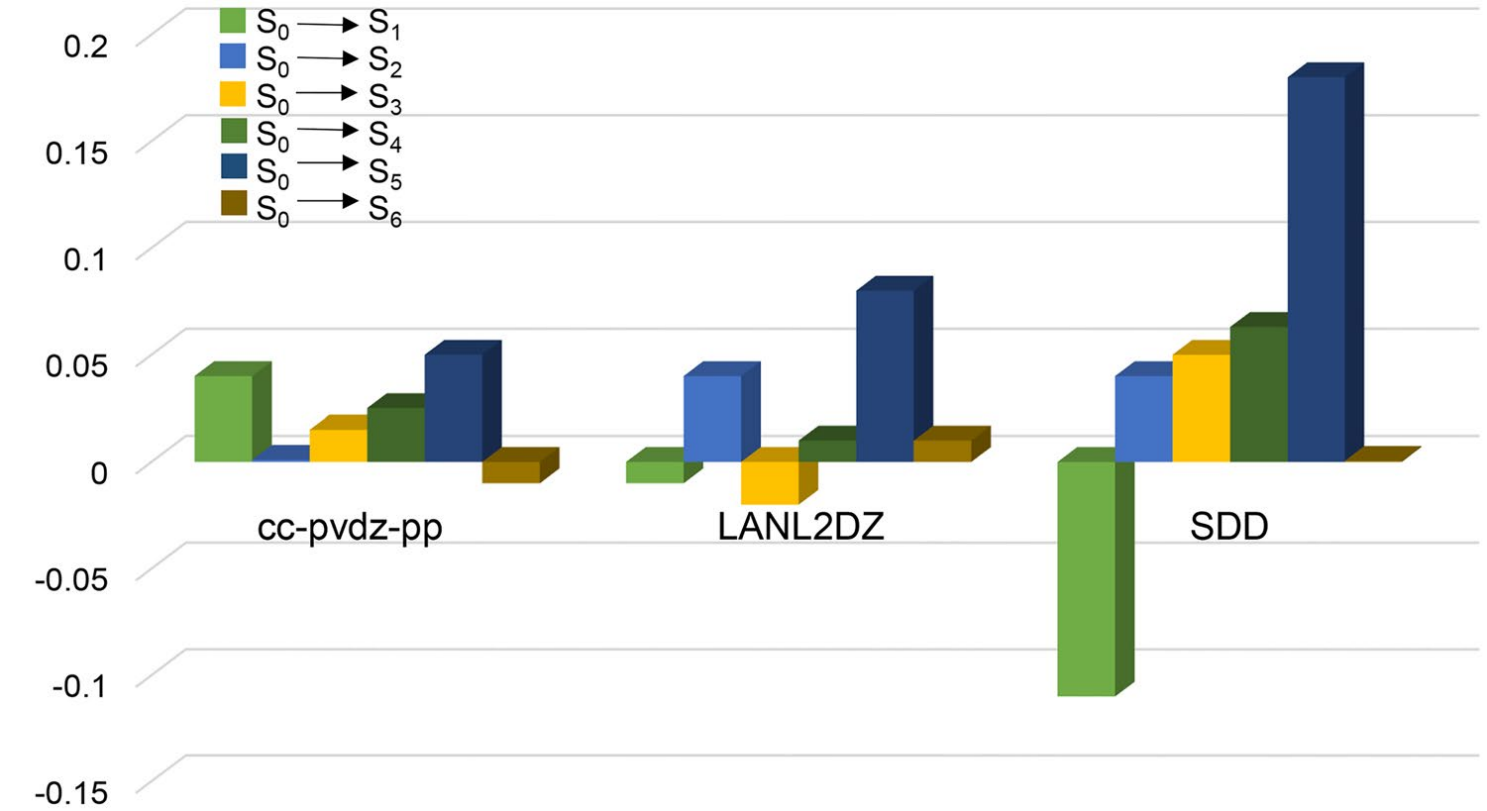
Errors of the first six excited state transition energies between aug-cc-pVDZ-pp and other three basis sets for Cd_2_S_2._

The errors of the first six excited state transition energies for Cd_2_S_2_ between cc-pVDZ-pp, LANL2DZ, SDD and the more precise aug-cc-pVDZ-pp basis sets are as follows: SDD > LANL2DZ > cc-pVDZ-pp, as shown in Fig. 3. The error of SDD is the largest, and those of cc-pVDZ-pp and LANL2DZ are not much different. Although the error of cc-pVDZ-pp is a little smaller than that of LANL2DZ, considering the higher calculation efficiency of LANL2DZ than that of cc-PVDZ-pp, the LANL2DZ is chosen in the calculation of Cd clusters. According to the above analysis, the *σ*_TPA_ value of the II–VI group semiconductors nano clusters were quantified by CAM-B3LYP. The Zn atoms used 6-31G*, and LANL2DZ was used for the Cd atoms.

**Figure 4:**
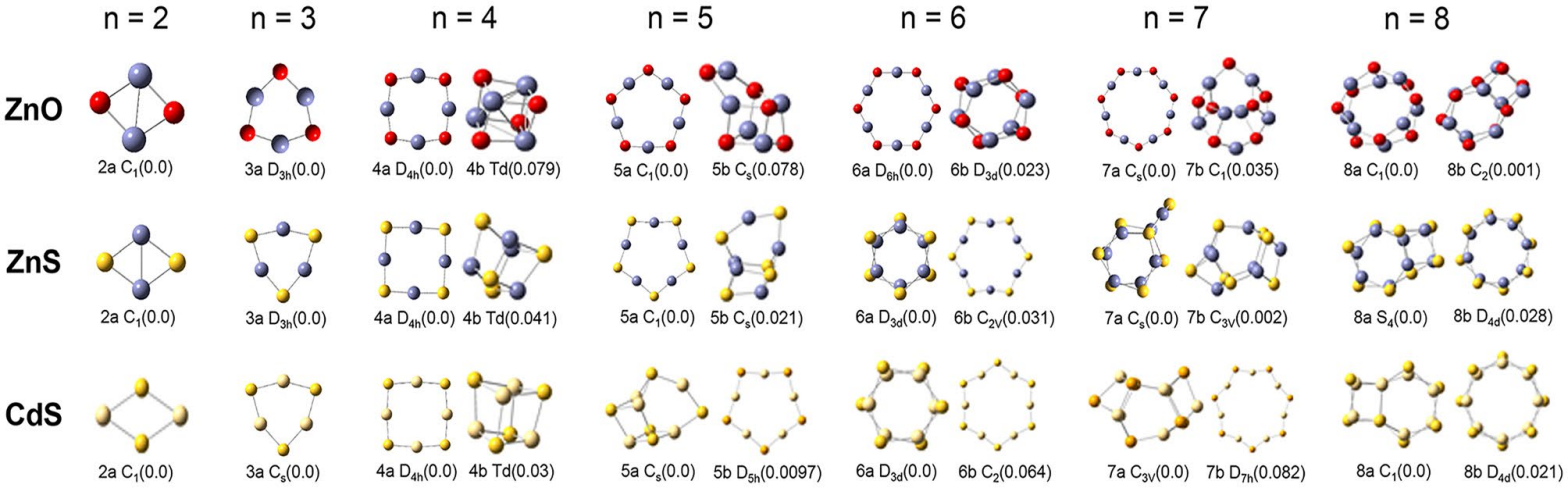
Optimized structures of Zn_n_O_n_, Zn_n_S_n_, and Cd_n_S_n_, n = 2–8 at the lowest/lower energy states. The energy from low to high is labeled by a and b. The values in the brackets are the energy difference to the lowest energy state of the corresponding structure.

The possible stable isomers of nano clusters with different size have been researched in the potential energy surface (PES). For NLO calculation, only isomers with lowest energy as shown in Fig. 4 have been considered. Table 3 gives the symmetry, bond length, bond angle, and energy gap between HOMO and LUMO of Zn_n_O_n_, Zn_n_S_n_, and Cd_n_S_n_, n = 2–8 at their lowest energy structures. In present work, the stable configurations on PES are consistent with previous study^46^. For Zn_n_O_n_ (n = 2–8) with smaller atomic numbers, the framework changes from two-dimensional (2D) to 3D when n = 8. With regard to the 2D framework, the bond length of Zn–O ranges from 1.77 Å to 1.89 Å, the bond angle of –O–Zn–O– ranges of ranges from102.70° to 179.38°, and ranges from77.30° to 126.10° for –Zn–O–Zn–. In addition, the bond length tends to decrease, while the bond angle tends to increase with increasing the number of n due to relaxation of ring tension. The range of HOMO–LUMO energy gap is 4.37 to 4.72 eV. The value of the HOMO–LUMO energy gap is larger in 2D structures than that in 3D structure. The unusual HOMO–LUMO energy gap comes from Zn_2_O_2_ with the value 2.70 eV, suggesting that it may appear properties distinguished from the other 2D structures.

**Table 3:** The symmetry, bond length, bond angle, and energy gap between HOMO and LUMO (*E*_g_) of Zn_n_O_n_, Zn_n_S_n_, and Cd_n_S_n_, n = 2–8 at their lowest energy structures.

n	Symmetry	Zn–O (Å)	–O–Zn–O– (°)	–Zn–O-Zn– (°)	*E*_g_ (eV)
2	C_1_	1.89	102.70	77.30	2.70
3	D_3h_	1.83	145.10	94.90	4.37
4	D_4h_	1.80	164.66	105.34	4.60
5	C_1_	1.79	174.17	113.91	4.72
6	D_6h_	1.78	179.38	120.62	4.59
7	C_s_	1.77	177.58	126.10	4.68
8	C_1_	1.87 to 2.15	92.95 to 151.28	84.99 to 118.61	4.01

		Zn–S(Å)	–S–Zn–S– (°)	–Zn–S–Zn– (°)	*E*_g_ (eV)
2	C_1_	2.27	113.87	66.13	2.77
3	D_3h_	2.21	157.16	82.84	4.17
4	D_4h_	2.18	176.63	93.44	4.46
5	C_1_	2.18	173.34	101.34	4.46
6	D_3d_	2.31 to 2.47	101.67 to 140.13	73.92 to 95.46	3.87
7	C_s_	2.15 to 2.46	100.15 to 139.90	73.38 to 109.50	3.49
8	S_4_	2.29 to 2.43	100.36 to 137.03	72.98 to 104.63	4.05

		Cd–S(Å)	–S–Cd–S– (°)	–Cd–S–Cd– (°)	*E*_g_ (eV)
2	C_1_	2.71	55.29	124.72	1.81
3	C_s_	2.48	152.77	87.29	3.33
4	D_4h_	2.46	172.61	97.39	3.45
5	C_s_	2.55	102.01 to 126.65	76.83 to 94.88	2.63
6	D_3d_	2.59 to 2.75	98.65 to 137.79	78.21 to 100.23	3.18
7	C_3V_	2.65	110.41 to 125.25	78.89 to 104.61	2.96
8	C_1_	2.60	49.54 to 134.17	79.40 to 108.42	3.18

In the case of Zn_n_S_n_ (n = 2–8), maintaining the elements of IIB group but augmenting the atomic number of the VIA element, the bond length, bond angle, and energy gap between HOMO and LUMO have different characters from those in Zn_n_O_n_ (n = 2–8). Due to the larger atomic radius of S, the stable clusters change from 2 to 3D when n = 6. As increasing the atomic number of the IIB elements further, the stable clusters begin to stay in the form of 3D frameworks when n = 5 in Cd_n_S_n_ (n = 2–8). To sum up, the larger the radius of atoms, the more stable of the clusters in their 3D forms. The HOMO–LUMO energy gaps of Zn_n_O_n_, Zn_n_S_n_, and Cd_n_S_n_, n = 2–8 in their 2D forms are larger than those in their 3D forms, except n = 2. For smallest cluster (n = 2), due to the delocalization of electrons in the whole molecule, the molecular energy gap becomes particularly small.

**Figure 5:**
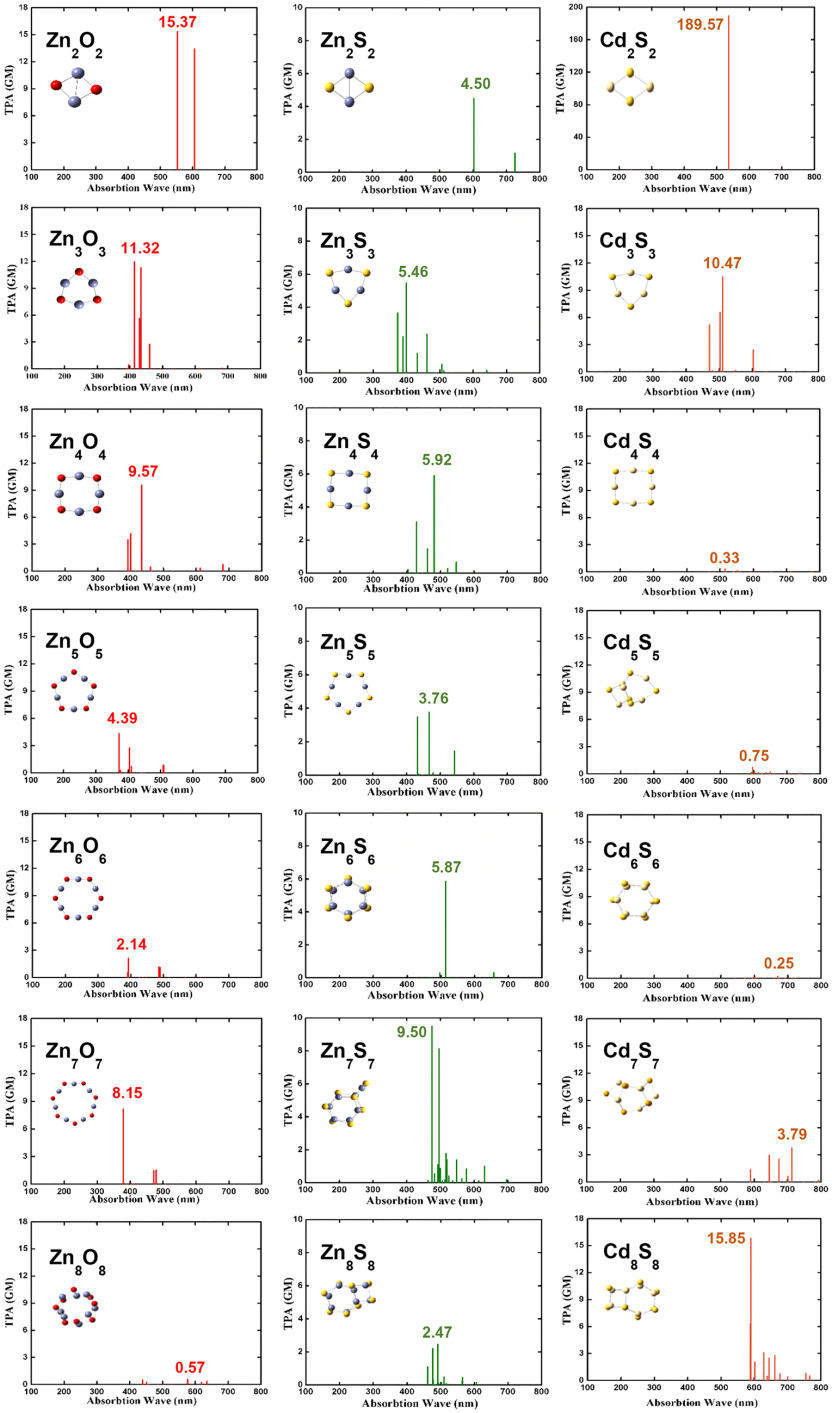
Calculated TPA cross sections of Zn_n_O_n_, Zn_n_S_n_, and Cd_n_S_n_, n = 2–8.

The calculated TPA cross sections of Zn_n_O_n_, Zn_n_S_n_ and Cd_n_S_n_, n = 2–8 are shown in Table 4 and Fig. 5. For the planar clusters of Zn_n_O_n_, the largest value of *σ*_TPA_ comes from Zn_2_O_2_ with the value 15.37 GM at 552.30 nm. The TPA cross section decreases with n increasing from 2 to 6, and the value drops to 2.14 GM when n = 6. However, the value of *σ*_TPA_ is enhanced to 8.15 GM at n = 7, the junction between the 2D and 3D structure. For Zn_n_S_n_ clusters of different sizes, their two-photon absorption cross sections vary from 2.47 to 9.50 GM. According to simple model as following in Eq. (5)^47^, the two-photon absorption cross section is inversely proportional to the square of the transition energy of first excited state and directly proportional to the transition matrix element. As shown in Table S1, all Zn_n_S_n_ clusters have similar first excitation energy, thus their two-photon absorption cross sections do not differ much.

**Table 4:** The TPA cross section *σ*_TPA_ (GM) and their corresponding maximum absorption wavelength λ_max_ (nm) of Zn_n_O_n_, Zn_n_S_n_, and Cd_n_S_n_, n = 2–8.

Zn_n_O_n_	2	3	4	5	6	7	8
*σ*_TPA_	15.37	11.32	9.57	4.39	2.14	8.15	0.57
λ_max_	552.30	414.72	434.33	372.37	393.65	379.20	576.74
Zn_n_S_n_	2	3	4	5	6	7	8
*σ*_TPA_	4.50	5.46	5.92	3.76	5.87	9.50	2.47
λ_max_	601.90	399.36	481.55	467.04	514.52	475.10	492.06
Cd_n_S_n_	2	3	4	5	6	7	8
*σ*_TPA_	189.57	10.47	0.33	0.75	0.25	3.79	15.85
λ_max_	536.80	510.29	515.59	596.15	670.27	712.64	589.07

5 $${\delta }_{TPA }\propto \frac{{M}_{01}^{2}{M}_{1n}^{2}}{({E}_{01}^{2}-{E}_{1n})}$$

Combined with the HOMO–LUMO energy gap mentioned above, Zn_n_O_n_, Zn_n_S_n_ and Cd_n_S_n_ nano clusters present excellent two-photon absorption properties due to planar and compact configuration leading to good delocalization of electrons. Especially, for Cd_2_S_2_, because cadmium and sulfur atoms have a larger radius and smaller electronegativity, valence electrons are more easily polarized thus it has a very large NLO response. Different from the 2D cases, the TPA cross sections of both Zn_n_S_n_ and Cd_n_S_n_ with 3D geometry show no obvious correlation with the number of n. The largest value of *σ*_TPA_ for Zn_n_S_n_ in the 3D case is 9.50 GM at 475.10 nm, from Zn_7_S_7_. On the other hand, the largest *σ*_TPA_ value for Cd_n_S_n_ is 15.85 GM at 589.07 nm, from Cd_8_S_8_.

By referring the symmetry of the 3D structures, the symmetry of Zn_7_S_7_ and Cd_8_S_8_ is C_s_ and C_1_, respectively, lower than the other corresponding 3D clusters. In other words, the symmetry has significant influence on the TPA of the 3D nano clusters, the lower the symmetry the higher the TPA cross section.

## Conclusions

Semiconductor clusters of Zn_n_O_n_, Zn_n_S_n_, and Cd_n_S_n_ (n = 2–8) were optimized and the corresponding stable structures were acquired. The symmetry, bond length, bond angle, and energy gap between HOMO and LUMO were analyzed. The results show that the larger the radius of atoms, the more stable of the clusters in their 3D forms. According to reasonable calculation and comparative analysis for Zn_2_O_2_, Zn_2_S_2_, and Cd_2_S_2_, CAM-B3LYP is more suitable for the calculating of the TPA cross sections for the II–VI semiconductor nano clusters, and LANL2DZ for the Cd atoms.

For the 2D nano clusters, sizes play important role on the TPA cross section. Generally, the value of TPA cross section will become abnormal at the junction between the 2D and 3D structures. In the case of the 3D nano clusters, the value TPA cross section are determined by the symmetries, the lower the symmetry the higher the TPA cross section.

## Supplementary Information

Supplementary Information.
