# Supplementary material for: Theoretical studies on the two-photon absorption of II–VI semiconductor nano clusters
Source: Sci Rep. 2022 Jan 7;12:110. doi: 10.1038/s41598-021-04203-w (PMC8742029; doi:10.1038/s41598-021-04203-w)
Supplement: Supplementary file 1 — Supplementary Information. [file 41598_2021_4203_MOESM1_ESM.docx]

Table S1. The TPA cross section *σ*_TPA_ (GM) and OPA cross section *σ*_OPA_ (GM), as well as their corresponding maximum absorption wavelength λ_max-TPA_ (nm) and λ_max-OPA_ (nm) of Zn_n_O_n_ and Zn_n_S_n_, n=2-8.

| Zn_n_O_n_ | 2 | 3 | 4 | 5 | 6 | 7 | 8 |
| --- | --- | --- | --- | --- | --- | --- | --- |
| *σ*_TPA_ | 15.37 | 11.32 | 9.57 | 4.39 | 2.14 | 8.15 | 0.57 |
| λ_max-TPA_ | 552.30 | 414.72 | 434.33 | 372.37 | 393.65 | 379.20 | 576.74 |
| *σ*_OPA_ | 0.27 | 0.12 | 0.14 | 0.17 | 0.25 | 0.00013 | 0.12 |
| λ_max-OPA_ | 280.54 | 207.29 | 228.41 | 224.36 | 213.67 | 194.63 | 288.35 |
| Zn_n_S_n_ | 2 | 3 | 4 | 5 | 6 | 7 | 8 |
| *σ*_TPA_ | 4.50 | 5.46 | 5.92 | 3.76 | 5.87 | 9.50 | 2.47 |
| λ_max-TPA_ | 601.90 | 399.36 | 481.55 | 467.04 | 514.52 | 475.10 | 492.06 |
| *σ*_OPA_ | 0.30 | 0.07 | 0.14 | 0.21 | 0.09 | 0.07 | 0.19 |
| λ_max-OPA_ | 246.13 | 267.22 | 211.78 | 213.96 | 257.01 | 241.02 | 240.86 |
